# High level of CD73 predicts poor prognosis of intrahepatic cholangiocarcinoma

**DOI:** 10.7150/jca.51038

**Published:** 2021-06-04

**Authors:** Ya-ping Xu, Ying-Qun Zhou, Yu-Jie Zhao, Yan Zhao, Feng Wang, Xiao-Yong Huang, Chuan-Yong Guo

**Affiliations:** 1 Department of Gastroenterology, Shanghai Tenth people's hospital, School of Clinical Medicine of Nanjing Medical University, Shanghai, 200072, P.R. China.; 2 Department of Gastroenterology, Shanghai Tenth People's Hospital, Tongji University School of Medicine, Shanghai, 200072, China.; 3 Liver Cancer Institute, Zhongshan Hospital, Fudan University; Key Laboratory of Carcinogenesis and Cancer Invasion (Fudan University), Ministry of Education, Shanghai 200032, P.R. China.

**Keywords:** CD73, CD8, intrahepatic cholangiocarcinoma, prognosis

## Abstract

**Background:** Despite recent improvements in the diagnosis and therapy of intrahepatic cholangiocarcinoma (ICC), the prognosis for ICC patients remains poor. Therefore, it is needed to identify new biological indicators for ICC progression.

**Methods:** Immunohistochemistry was engaged to inspect the ecto-5ʹ-nucleotidase (CD73) and CD8 expressions in tissue microarrays including tissues from 140 ICC patients. Then, the association between the level of CD73/CD8 and clinicopathologic characteristics of ICC was analysed. Finally, the prognostic value of CD73 and CD8 levels in ICC patients was assessed by Kaplan-Meier and multivariate and univariate analyses.

**Results:** The CD73 expression was evidently upregulated in ICC tissues compared to the corresponding peritumoral tissues. The elevated CD73 expression was positively related to the lymphatic metastasis (p=0.049). While the level of tumour-infiltrating CD8 T^+^ cells in tumour tissues was negatively associated with serum AFP (p=0.019), tumor size (p=0.028), and lymphatic metastasis (p=0.039). Additionally, patients with elevated CD73 expression or low tumour-infiltrating CD8^+^ T cells exhibited shorter overall survival (OS) and higher disease-free survival (DFS) rates than patients with low CD73 expression and/or high tumour-infiltrating CD8^+^ T cells. Notably, the overexpression of CD73 or low tumour-infiltrating CD8^+^ T cells was an independent indicator for predicting the OS and DFS of ICC patients.

**Conclusions:** We revealed that CD73 expression and low tumour-infiltrating CD8^+^T cells are valuable predictors of survival and recurrence in patients with ICC.

## Introduction

Intrahepatic cholangiocarcinoma (ICC) represents the 2^nd^ most common primary liver malignancy and is increasing in incidence 1. Despite advances have been made in the early diagnosis of ICC, the majority of patients are still diagnosed at a terminal stage, and only roughly 20% of patients have the chance for curative surgical resection. Furthermore, almost 80% patients may subsequently appear postoperative disease recurrence 2. Hence, an advance in comprehending the mechanisms of tumour recurrence will be profitable for ICC patients.

CD73 (cluster of differentiation 73 or ecto-5′-nucleotidase, NT5E) is a sort of lymphocytic differentiation antigen in diverse tissues and cell types 3. Functionally, CD73 participates in many vital physiological and pathophysiological signalling via adenosine receptors to catalyse extracellular 5′-adenosine monophosphate to adenosine, which serves as an essential regulator in the purinergic signalling pathway. Now, CD73 was illustrated to be critical not only in supervising the production of extracellular adenosine to regulate adenosine pathway but also in adjusting other biological processes, including the inflammation and immune response. For instance, CD73 has important effects on cancer cell proliferation and survival via reshaping the tumour immune microenvironment 4. Recently, CD73 was discovered to be widely expressed on numerous tumour cell lines and overexpressed in several cancerous samples such as colon cancer, mammary carcinoma, and prostate cancer 3,5,6. Moreover, CD73 was revealed to be a potential prognostic marker for patients with breast cancer and was found to promote breast cancer progression via regulate epidermal growth factor receptor (EGFR) level 7. In the liver, immunohistochemical and enzyme histochemical studies have shown that CD73 was distributed in the biliary canaliculi of hepatocytes and stellate cells and played an important role in the pathogenesis of hepatic fibrosis, which ultimately leaded to cancer 8. However, the role of CD73 in ICC remains unexplored.

Accumulating evidence indicated that the CD73 could be regarded as a novel immune checkpoint that induced cancer development through restraining the anti-tumour immune response. Here, we aimed to analyse CD73 expression in ICC tissues, and to explore the relationship between the CD73 expression and/or level of CD8^+^ T cells and the clinicopathological characteristics. Moreover, their prognostic significance in ICC patients was also investigated.

## Materials and methods

### Patients and following-up

Tumour and paired peritumoral samples were acquired from 140 ICC patients who underwent the surgical resection at Zhongshan Hospital, Fudan University from February 1999 to November 2006. The surgical resection was defined as our previous report 1. The histopathological diagnosis was independently determined by two pathologists. The ethical approval was obtained from the Research Ethics Committee and institutional review board of Zhongshan Hospital, and informed written consent was obtained from every patient. All following-up data were itemized in February 2009. The median following-up was 25 months (range 4 to 120 months). The following-up measures were delineated in our earlier study 1. The clinicopathological features of all patients were recorded in **Table 1**.

### Tissue Microarray Construction and Immunohistochemistry

The tissue microarray (TMA) was built as depicted previously 1. Briefly, all tissues were histologically explored by two pathologists via haematoxylin and eosin staining (HE) and pre-tagged the representing regions in paraffin blocks. About 1 mm diameter cylinders from 2 representative regions was obtained from every sample. Then, two TMA blocks (one is ICC, and another is a paired peritumoral) were built (Shanghai Biochip Co., Ltd., Shanghai), and each included 140 cylinders.

Rabbit anti-CD73 antibody (ab124725, Abcam) and rabbit anti-CD8 antibody (ab237709, Abcam) antibodies were employed to determine the CD73 and CD8 proteins. The intensity of positive staining was estimated as described 9. Briefly, the immunohistochemical staining was appraised by two pathologists independently. CD73 staining was marked according to the percentage of positively stained cells, 0, no positive cells; 1, ≤ 25%; 2, > 25 ~ ≤ 50%; 3, >50 ~ ≤ 75%; and 4, ≥ 75%. The 1 and 2 was classified as low expression (CD73^low^), while the 3 and 4 as high expression (CD73^high^) in ICC tissues.

CD8-positive cells in the photographs were appraised by the Leica Qwin Plus, and the number of CD8-positive cells in each photograph was used to represent each sample. The expression of CD8 was categorized into 2 subgroups based on intensity.

### Statistical Analysis

Statistical analysis was done with SPSS 19.0 (SPSS). The χ^2^ test and Fisher's exact test were utilized the difference between groups. Overall survival (OS) and the disease-free survival (DFS) were defined as previously reported 1. Prognostic significance was appraised using Kaplan-Meier and log-rank tests analyses. Cox's proportional hazards regression model was used to analyse the independent prognostic factors. The less than 0.05 of* p* value was deemed to be significant difference.

## Results

### Expression of CD73 was positively related to malignant phenotypes of ICC

**Figure 1:**
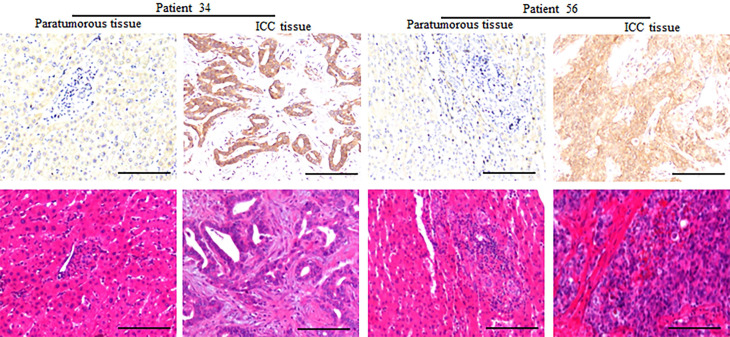
** Expression of CD73 protein in paratumorous normal liver and ICC tissues.** Representative images of CD73 in the corresponding normal liver and ICC, which showed that the expression of CD73 in ICC tissues is higher than that in the corresponding normal liver (Bar: 100 µm).

**Figure 2:**
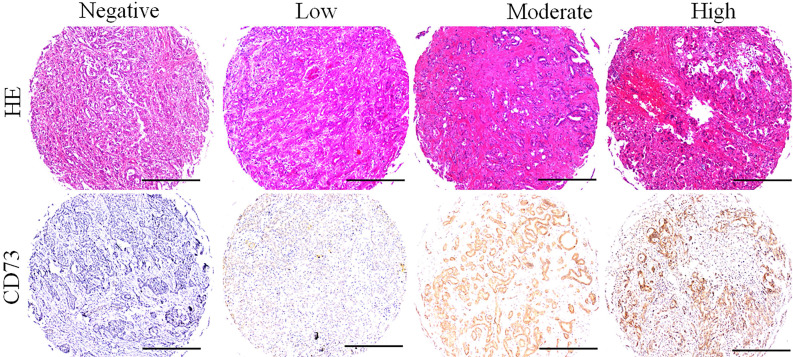
** Different level of CD73 protein in ICC tissues.** Representative images of different CD73 staining in ICC tissues, and the expression of CD73 in ICC tissues with heterogenicity (Bar: 50 µm).

The CD73 protein was resided in the cell cytoplasm, and upregulation in ICC tissues compared to paired peritumoral tissues (**Fig. 1**). The expression of CD73 displayed great heterogeneity among ICC tissues (**Fig. 2**). In ICC, CD73^high^ was observed in 48.5 % (68/140) of all tumour samples. As shown in **Table 1**, CD73^high^ was positively correlated with lymphatic metastasis (p=0.049), whereas other clinical features, containing age, sex, HBsAg background, liver cirrhosis, preoperative serum carbohydrate antigen 19-9 (CA19-9) and Child-Pugh score, were not significantly related to the expression of CD73.

### Level of tumour-infiltrating CD8^+^ T cell was positively associated with malignant phenotypes of ICC

The staining of CD8 was found in ICC and paired peritumoral tissues with diversity, and more CD8 staining in paired peritumoral tissues compared to ICC tissues. The CD8 protein resided in the cell cytoplasm, Fifty-eight of all ICC tissues displayed a high level of tumour-infiltrating CD8 (42.0%, 58/140). Patients with low levels of tumour-infiltrating CD8 were more likely to exhibit aggressive features. Tumour-infiltrating CD8^low^ patients harboured high Serum AFP (p = 0.019), large tumor size (p = 0.028), and Lymphatic metastasis (p = 0.039) (**Table 1**).

### Elevated CD73 and/or low tumour-infiltrating CD8 T cells were independent parameters for predicting the prognosis of ICC patients

**Figure 3:**
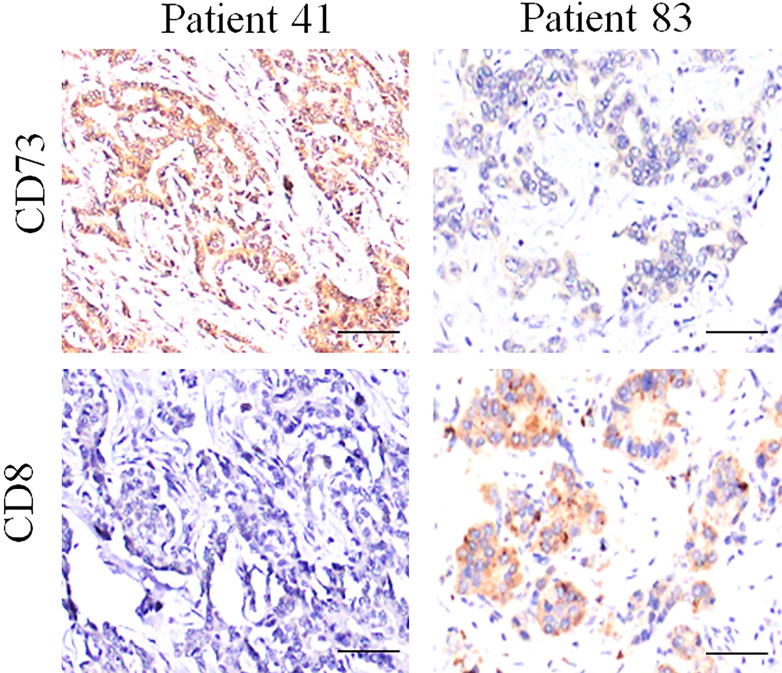
** Expression of CD73 and CD8 protein in ICC tissues.** Representative images of tumor tissues with different CD73 and CD8 staining, which showed that the CD73 expression is negatively associated with the CD8 staining.

Several reports have demonstrated that the elevated expression of CD73 depressed antitumor immunity by impairing the infiltration and function of CD8^+^T cell; however, we did not find the obviously negative relationship between CD8 and CD73 staining in ICC tissues (**Fig. 3 and Fig. S1**).

**Figure 4:**
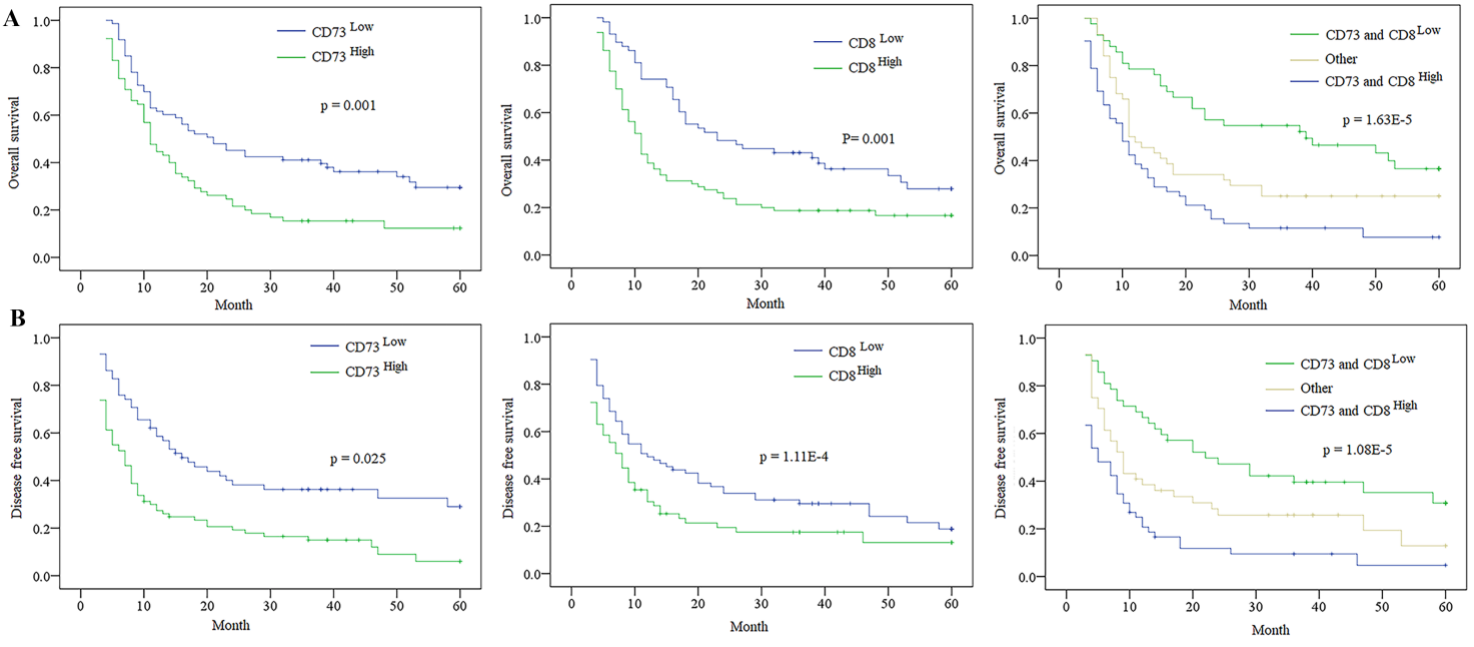
** The Expression of CD73 and/ or CD8 in predicting the prognosis of ICC patients. A.** Kaplan-Meier analysis of OS for CD73, CD8 and combination of CD73 and CD8 level in ICC. **B.** Kaplan-Meier analysis of DFS for CD73, CD8 and combination of CD73 and CD8 level in ICC.

At the last following-up, 108 cases appeared tumour relapse, and 109 cases died, including seven cases without tumour relapse. The 2- and 5-year OS and DFS rates in the whole population were 77.9% and 70.0%, 35.7% and 23.6%, respectively. The univariate analysis revealed that massive tumour (>5 cm), poor differentiation, multiple tumours, microvascular/bile duct invasion, and lymphatic metastasis were predictors of OS and DFS. Other features, containing age, sex, and history of hepatitis, had no prognostic significance for OS and DFS (**Table 2**). The expression of CD73 was disclosed to be related to the OS and DFS rates (**Table 2**). The 5-year OS in the CD73^low^ group was evidently higher than that in the CD73^high^ group (p= 0.001, **Fig. 4**). The 5-year DFS rate in the CD73^low^ group were distinctly lower than that in the CD73^high^ group (p= 0.025, **Fig. 4**). The expression of CD8 was also obviously correlated with the OS and DFS rates (**Table 2**). The postoperative 5-year OS rate of ICC patients was higher in the CD8^high^ group than that in the CD8^low^ group (*p* =0.001), and the DFS rate of ICC patients in the CD8^low^ group were clearly higher than those in the CD8^high^ group (*p* = 1.11E-4) (**Fig. 4**).

We then investigated the combined role of CD73 and the level of tumour infiltrating CD8^+^T cells in survival. We categorized all cases into three subgroups according to the CD73 and CD8 staining: group I, high CD73 and low CD8^+^T cells; group II, both high CD73 and tumour infiltrating CD8^+^ T cells or both low CD73 and tumour infiltrating CD8^+^T cells; and group III, low CD73 and high tumour infiltrating CD8^+^ T cells. The 5-year OS rate in group I were evidently higher than those in groups II and III. The 5-year DFS rate in group I were distinctly lower than those in groups II and III (**Fig. 4**). The multivariate Cox proportional hazards model disclosed that the CD73 expression, tumour number, and level of both CD73 and CD8 were independent prognostic indicators for OS and DFS; however, the level of tumour infiltrating CD8^+^ T cells was not an independent prognostic indicator for DFS (**Table 2 and 3**).

## Discussion

ICC is a destroying disease with limited therapy choices and poor prognosis. Although surgical resection is limited to patients with early-stage disease, this method still is the merely curative therapeutic mean for ICC patients. Presently, chemotherapy is the only confirmed standardized experience for the majority of ICC patients in advanced stages. Accordingly, there is a practical requirement to advance fresh therapies for this disease. CD73, initially characterized as a lymphocyte differentiation antigen, has been revealed to be a co-signalling molecule on T cells and an adhesion molecule that is required for lymphocytes to adhere to the endothelium 10,11. Recently, it was revealed that elevated CD73 expression on cancer cells damages the anti-tumour T cell response 10. We showed here that CD73 expression was upregulation in almost half of our ICC tissues, and the expression of CD73 was positively correlated with the lymphatic metastasis of ICC. The CD8 staining was low in ICC tissues compared to paired peritumoral tissues, and patients with low tumour-infiltrating CD8 T cells often accompanied with Serum AFP, large tumor size and Lymphatic metastasis. Moreover, we showed that the CD73 expression and level of tumour-infiltrating CD8^+^T cells were independent indexes for predicting the prognosis of ICC patients. Thus, a high level of CD73 or low tumour-infiltrating CD8^+^T cells serves as an index of ICC progression.

Previous studies have reported that CD73 is upregulated in and promotes the progression of various human cancers, containing colon, lung, pancreas, breast and ovary cancer 3,12-14. For instance, a high level of CD73 was found to promote tumour angiogenesis, invasion, and metastasis and was associated with a short OS time in breast cancer^15^. The overexpression of CD73 was found in highly invasive human melanoma cells 16, but not in melanocytes and primary tumour cells; moreover, CD73 has been demonstrated to serve as a proliferative factor for glioma cells. Additionally, elevated CD73 expression was suggested to be a prognostic marker for patients with colon cancer 3. Here, we also found that CD73 expression was elevated in ICC tissues compared to paired peritumoral tissues. Furthermore, we discovered that the expression of CD73 was positively associated with the malignant phenotype of ICC, and a high level of CD73 was correlated with the low OS and high DFS rates in ICC patients. Moreover, the cox analysis uncovered that CD73 expression is an independent prognostic marker for the OS of ICC patients. Thus, we presumed the high level of CD73 fostered ICC development and might serve as a prognostic biomarker for the OS of ICC patients.

Recent studies have shown that cancer exosomes express CD39 and CD73, which suppress T cells through adenosine production. Furthermore, CD73 is an ectonucleotidase that cooperates in the extracellular adenosine production through ATP hydrolysis to attack the balance of immunosuppressive microenvironments, indicating that CD73 could depress the CD8^+^T cell responses 17-20. Here, we found that a weak negative relationship between the CD73 level and tumour infiltrating CD8^+^T cells, which might indicate that the tumour infiltrating CD8^+^T cells might be impacted by other factors. The cox analysis showed the combined CD73 expression and tumour infiltrating CD8^+^T cells could be deemed as a powerful prognostic indicator for the OS and DFS of patients with ICC. Now, emerging evidence demonstrated the immunosuppressive function of CD73 in tumour, and the inhibitory action of the anti-CD73 antibody on breast cancer growth and metastasis, the high level of CD73 might not only serve as an important prognostic index but could also be a potential therapeutic target in ICC.

In conclusion, we have provided that a high level of CD73 foster ICC development, and CD73 level and/or tumour infiltrating CD8^+^T cells can be the important biomarkers for the prognosis of ICC patients.

## Supplementary Material

Supplementary figure S1.

## Figures and Tables

**Table 1:** Correlations between CD73 or CD8 and clinicopathological features in 140 ICC

Variables	CD73^low^	CD73^high^	*P* value	CD8^high^	CD8^low^	*P* value
**Age (years)**						
≥ 53	33	37	0.499	33	37	0.865
<53	37	33		32	38	
**Sex**						
Male	25	34	0.171	25	34	0.412
Female	45	36		40	41	
**HBsAg**						
Positive	41	46	0.384	42	45	0.574
Negative	29	24		23	30	
**Liver cirrhosis**						
Yes	26	31	0.390	25	32	0.614
No	44	39		40	43	
**Serum CA19-9 (ng/ml)**						
≥37	44	41	0.604	41	44	0.594
<37	26	29		24	31	
**Serum ALT (U/l)**						
≥75	12	7	0.217	9	10	0.930
<75	58	63		56	65	
**Child-Pugh score**						
A	71	63	0.511*	63	71	0.686*
B	4	2		2	4	
**Serum AFP (ng/ml)**						
<20	63	59	0.313	52	70	0.019
≥20	7	11		13	5	
**Tumor size (diameter, cm)**						
≤5	57	52	0.309	56	53	0.028
>5	13	18		9	22	
**Tumor differentiation**						
III/IV	41	31	0.091	34	34	0.410
I/II	29	39		31	41	
**Tumor number**						
Multiple	5	6	0.753	4	7	0.517
Single	65	64		42	87	
**Microvascular/bile duct invasion**						
Yes	8	15	0.110	4	7	0.544*
No	62	55		61	68	
**Lymphatic metastasis**						
Yes	12	22	0.049	21	13	0.039
No	58	48		44	62	

**Abbreviations and Note:** ICC, intrahepatic cholangiocarcinoma; AFP, alpha-fetoprotein; HBsAg, hepatitis B surface antigen; X^2^ test. CD73^high^, ≥50% of tumor section, and CD73^low^, <50%. *Fisher's Exact Test.

**Table 2:** Univariate and multivariate analyses of factors associated with survival of ICC patients

Factors	OS			
	Univariate	Multivariate		
	*P*	HR	95% CI	*p*
Age, Years (<53 *vs.*≥53)	0.450			NA
Sex (male *vs.* female)	0.959			NA
HBsAg (negative* vs.* positive)	0.739			NA
Liver cirrhosis (yes *vs.* no)	0.427			NA
Child-Pugh score (A *vs.* B)	0.125			NA
Serum ALT (<75 *vs.* ≥75, U/l)	0.618			NA
Serum CA19-9 (<37 *vs.* ≥37, ng/ml)	0.387			NA
Serum AFP (<20 *vs.* ≥20, ng/ml)	0.241			NA
Tumor size (≤5 cm *vs.* >5cm)	0.025			NS
Tumor differentiation (I/II *vs.* III/IV)	0.043	0.669	0.453-0.987	0.043
Tumor number (multiple *vs.* single)	0.021	0.438	0.227-0.846	0.014
Microvascular/bile duct invasion (yes* vs.* no)	0.031			NS
Lymphatic metastasis (no *vs.* yes)	0.007			NS
CD73 (high *vs.* low)	0.009	1.632	1.091-2.440	0.017
CD8 (high *vs.* low)	0.010	0.634	0.426-0.945	0.025
Combine of and Group I *vs.* Group II and III	<0.001	0.612	0.490-0.766	<0.001

Abbreviations and Note: OS, overall survival; NA, not adopted; NS, not significant; AFP, alpha-fetoprotein; HBsAg, hepatitis B surface antigen; 95%CI, 95% confidence interval; HR, Hazard ratio; Cox proportional hazards regression model. *Group I, CD73^high^ and CD8^low^; Group II, both high or both low; Group III, CD73^low^ and CD8^high^.

**Table 3:** Univariate and multivariate analyses of factors associated with recurrence of ICC patients

Factors	OS, Cumulative recurrence			
	Univariate	Multivariate		
	*P*	HR	95%CI	*p*
Age, Years (<53 *vs.*≥53)	0.308			NA
Sex (male *vs.* female)	0.742			NA
HBsAg (negative* vs.* positive)	0.366			NA
Liver cirrhosis (yes *vs.* no)	0.652			NA
Child-Pugh score (A *vs.* B)	0.073			NA
Serum ALT (<75 *vs.* ≥75, U/l)	0.782			NA
Serum CA19-9 (<37 *vs.* ≥37, ng/ml)	0.314			NA
Serum AFP (<20 *vs.* ≥20, ng/ml)	0.764			NA
Tumor size (≤5cm *vs.* >5cm)	0.006	0.513	0.296-0.890	0.018
Tumor differentiation (I/II *vs.* III/IV)	0.032			NS
Tumor number (multiple *vs.* single)	0.002	0.455	0.236-0.877	0.019
Microvascular/bile duct invasion (yes* vs.* no)	0.030			NS
Lymphatic metastasis (no *vs.* yes)	0.001	0.626	0.406-0.965	0.034
CD73 (high *vs.* low)	0.029	1.451	0.968-2.174	0.071
CD8 (high *vs.* low)	0.049	0.808	0.545 -1.197	0.228
Combine of and Group I *vs.* Group II and III	<0.001	0.747	0.598-0.934	0.010

Abbreviations and Note: OS, overall survival; NA, not adopted; NS, not significant; AFP, alpha-fetoprotein; HBsAg, hepatitis B surface antigen; 95%CI, 95% confidence interval; HR, Hazard ratio; Cox proportional hazards regression model. *Group I, CD73^high^ and CD8^low^; Group II, both high or both low; Group III, CD73^low^ and CD8^high^.

## Abbreviations

ICC: intrahepatic cholangiocarcinoma
CD73: ecto-5′-nucleotidase
IHC: immunohistochemistry
TMA: tissue microarray
HE: haematoxylin and eosin staining
AFP: alpha-fetoprotein
CA19-9: carbohydrate antigen 19-9
DFS: disease free survival
OS: Overall survival
