# Supplementary material for: High level of CD73 predicts poor prognosis of intrahepatic cholangiocarcinoma
Source: J Cancer. 2021 Jun 4;12(15):4655–60. doi: 10.7150/jca.51038 (PMC8210563; doi:10.7150/jca.51038)
Supplement: Supplementary file 1 — Supplementary figure S1. [file jcav12p4655s1.pdf]

Supplementary

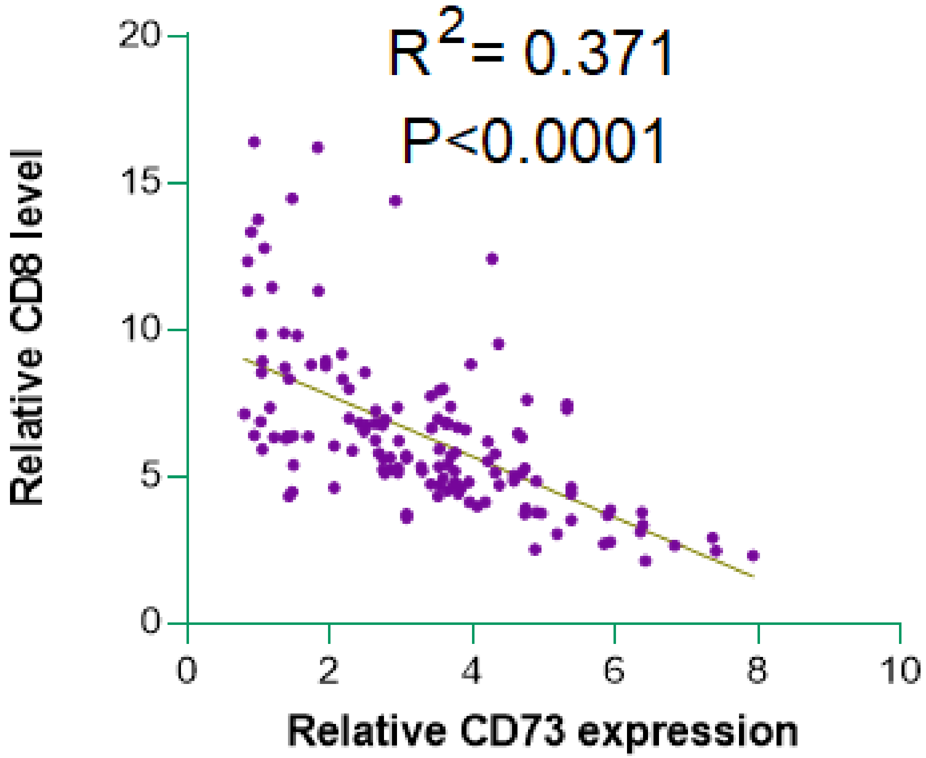

Figure s1. The relation between CD73 and CD8 staining in ICC tissues. The CD73 expression was weakly negatively associated with the CD8 staining in ICC tissues.
